# Factors associated with postoperative renal sinus invasion and perinephric fat invasion in renal cell cancer: treatment planning implications

**DOI:** 10.18632/oncotarget.23497

**Published:** 2017-12-15

**Authors:** Dong Ni, Xin Ma, Hong-Zhao Li, Yu Gao, Xin-Tao Li, Yu Zhang, Qing Ai, Qing-Bo Huang, Jun-Yao Duan, Xu Zhang

**Affiliations:** ^1^ Department of Urology, Chinese PLA General Hospital, Kidney Disease State Key Laboratory of Chinese PLA General Hospital, Beijing, 100853, China; ^2^ Department of Urology, Union Hospital, Tongji Medical College, Huazhong University of Science and Technology, Wuhan, 430022, China

**Keywords:** renal cell carcinoma, renal sinus invasion, perinephric fat invasion, risk factors, risk stratification

## Abstract

In patients with renal cell carcinoma (RCC), postoperative upstaging including perinephric fat invasion (PNI) and renal sinus invasion (RSI) leads to unfavorable oncological outcomes. Determining the preoperative risk factors for postoperative upstaging could be beneficial for treatment planning. In this study, 267 RCC patients who underwent radical nephrectomy were studied retrospectively. The RSI incidence was significantly greater than that of PNI. Kaplan-Meier analysis revealed that patients with RSI, PNI, and RSI plus PNI had poorer disease-free-survival than those with neither RSI nor PNI. Univariate and multivariate logistic regression analyses indicated that a tumor extension into the sinus, an irregular tumor-sinus border, and an irregular tumor shape in CT/MRI imaging were independent risk factors for RSI. And a tumor larger than 5 cm, an irregular tumor-perinephric fat border, and a tumor necrosis were independent risk factors for PNI. Subgrouping of patients into low-, moderate-, and high-risk groups according to these factors, revealed a direct association between the risk factors and PNI/RSI incidence. In conclusion, in patients with RCC, preoperative risk factors associated with postoperative upstaging could be assessed by imaging data obtained using CT or MRI. Preoperative Risk group classification would be clinically useful for patient counseling and treatment planning.

## INTRODUCTION

Renal cell carcinoma (RCC) accounts for almost 3% of all human cancers [1] ,and its incidence is increasing. The prognoses and treatments differ considerably between localised (T1–2N0M0) and advanced RCC. The 5-year cancer-specific survival rates are > 70% and < 54.7% for localised and advanced RCC, respectively [2]. According to the European Association of Urology (EAU) guidelines, nephron-sparing surgery (NSS) is regarded as the first choice in treating localised masses [3]. With advances in surgical techniques and instruments, larger, more complex cases, such as centralized RCC, are no longer a barrier to NSS [4]. However, pathological uncertainty exists regarding localised renal tumors, and there is a postoperative risk of upstaging to pT3a, which predominately arises because of renal sinus invasion (RSI) and perinephric fat invasion (PNI) [5–7]. Additionally, pathological upstaging was reported to have worse oncological outcomes in RCC patients [6, 8].

The renal sinus is located between the pelvicalyceal system and renal parenchyma, and consists of fat tissue, lymphatics, and numerous renal vein tributaries [9]. In addition, there is no fibrous barrier to delineate the renal sinus from the parenchyma, whereas the perinephric fat tissue and renal parenchyma are separated by a fibrous capsule. Therefore, theoretically, RSI is more likely to occur than PNI. In this retrospective study, we compared the incidence and prognostic significance of postoperative RSI and PNI in patients with RCC who underwent radical nephrectomy (RN). Moreover, we aimed to identify preoperative risk factors associated with RSI and PNI development in such patients.

## RESULTS

### RSI had a higher incidence than PNI

A total of 267 patients with RCC were enrolled in this study. The most common RCC subtype (90.2%) was clear cell RCC. The tumor size ranged from 1 to 13 cm (mean ± standard deviation (SD), 5.2 ± 2.1 cm). A total of 60 patients were confirmed to have postoperative upstaging. Specifically, RSI was identified in 45 patients and PNI was identified in 25 patients. Twelve patients had both RSI and PNI. Two patients identified only small renal vein invasion. The incidence of RSI was significantly higher than that of PNI (Pearson χ^2^ test, *p* = 0.014). The patients’ clinicopathological characteristics are shown in Table 1.

**Table 1:** Clinicopathological characteristics of the study population

Characteristics	Study population (*n* = 267)
Age	57.3 ± 11.6
Sex	
Male	188 (70.4)
Female	79 (29.6)
BMI	25.7 ± 3.2
Tumor size (cm)	5.2 ± 2.1
Histology	
Clear cell	241(90.2)
Chromophobe	3 (1.1)
Papillary	8 (3.0)
Collecting ducts carcinoma	1 (0.4)
Oncocytoma	6 (2.2)
Translocation RCC Xp11.2	5 (1.9)
Mixed type	1(0.4)
Other	2 (0.7)
Furhman grade	
I	4 (1.5)
II	166 (62.2)
III	58 (21.7)
IV	16 (6.0)
Tumor stage	
T1a	94 (35.2)
T1b	94 (35.2)
T2a	17 (6.4)
T2b	2 (0.7)
T3a	60 (22.5)
PNI	13
RSI	33
PNI+RSI	12
Vein invasion only^a^	2

Data were presented as the mean ± SD or number (percentage).

^a^Eighteen patients had renal vein segment invasion. Simultaneous RSI and/or PNI were present in 16 patients. Two patients had renal vein segment invasion alone.

BMI, body mass index; PNI, perinephric fat invasion; RSI, renal sinus invasion.

### Short term oncological outcomes

**Figure 1:**
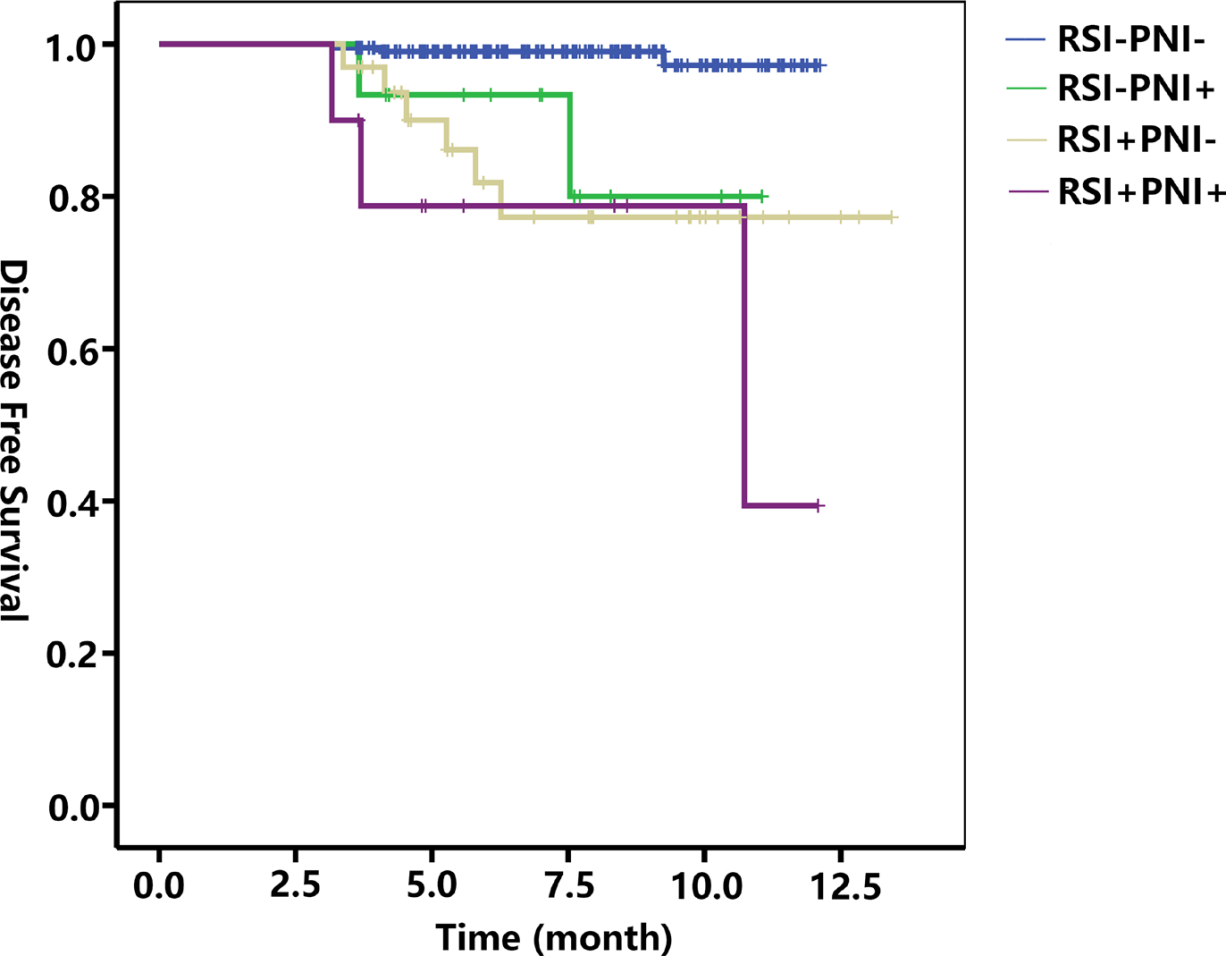
Kaplan-Meier analysis of disease-free survival Patients with RSI, PNI, and RSI plus PNI had poorer DFS than those with neither RSI nor PNI. Log-rank test: RSI+PNI-, p < 0.001; RSI-PNI+, p = 0.002; RSI+PNI+, p < 0.001 vs. RSI-PNI-, respectively. No significant differences were identified in the prognoses between patients with RSI, PNI, or both. Log-rank test: RSI vs PNI, p = 0.673; RSI vs RSI+PNI+, p = 0.352; PNI vs RSI+PNI+, p = 0.424. DFS: disease-free survival; RSI: renal sinus invasion; PNI: perinephric fat invasion

At the end point of this study, the recurrence rates were 21.2% (7/33) for patients with RSI alone, 7.7% (1/13) for patients with PNI alone, and 33.3% (4/12) for patients with RSI plus PNI. However, the differences between the groups were not significant. As shown in Figure 1, Kaplan-Meier analysis revealed that patients with RSI, PNI, and RSI plus PNI had poorer disease-free-survival (DFS) than those with neither RSI nor PNI (log-rank test, RSI+PNI- *p* < 0.001; RSI-PNI+ *p* = 0.002; RSI+PNI+ *p* < 0.001 vs RSI-PNI-, respectively). However, there were no significant differences in the prognoses between patients with RSI, PNI, or both (all *p* > 0.05).

### Risk factors associated with RSI

Univariate analysis revealed that large tumors (> 5 cm; *p* = 0.045), tumor extension into the sinus (*p* = 0.022), an irregular tumor-sinus border (*p* < 0.001), tumor necrosis (*p* < 0.001), and an irregular tumor shape (*p* < 0.001) were preoperative factors associated with RSI (Table 2).

**Table 2:** Univariate and multivariate logistic regression analyses of risk factors for predicting RSI

Factors	Univariate analysis				Mutivariate analysis		
	HR	95% CI	*p*		HR	95% CI	*p*
SEX	0.842	0.442–1.603		0.601			
Age	1.010	0.983–1.036		0.480			
BMI	1.059	0.968–1.159		0.208			
Tumor size (cm)							
≤ 5	1				1		
> 5	1.865	1.015–3.427		0.045	2.151	0.858–5.376	0.103
Imaging extend to sinus							
No	1				1		
Yes	10.500	1.407–78.356		0.022	10.723	0.243–92.501	0.031
Necrosis							
No	1				1		
Yes	3.750	1.923–7.313		< 0.001	1.916	0.842–4.306	0.121
Irregular border							
No	1				1		
Yes	0.044	0.017–0.117		< 0.001	0.066	0.023–0.191	< 0.001
Irregular shape							
No	1				1		
Yes	0.166	0.081–0.340		< 0.001	0.356	0.014–0.896	0.028

RSI, renal sinus invasion; BMI, body mass index; HR, hazard ratio; CI, confidence interval.

**Figure 2:**
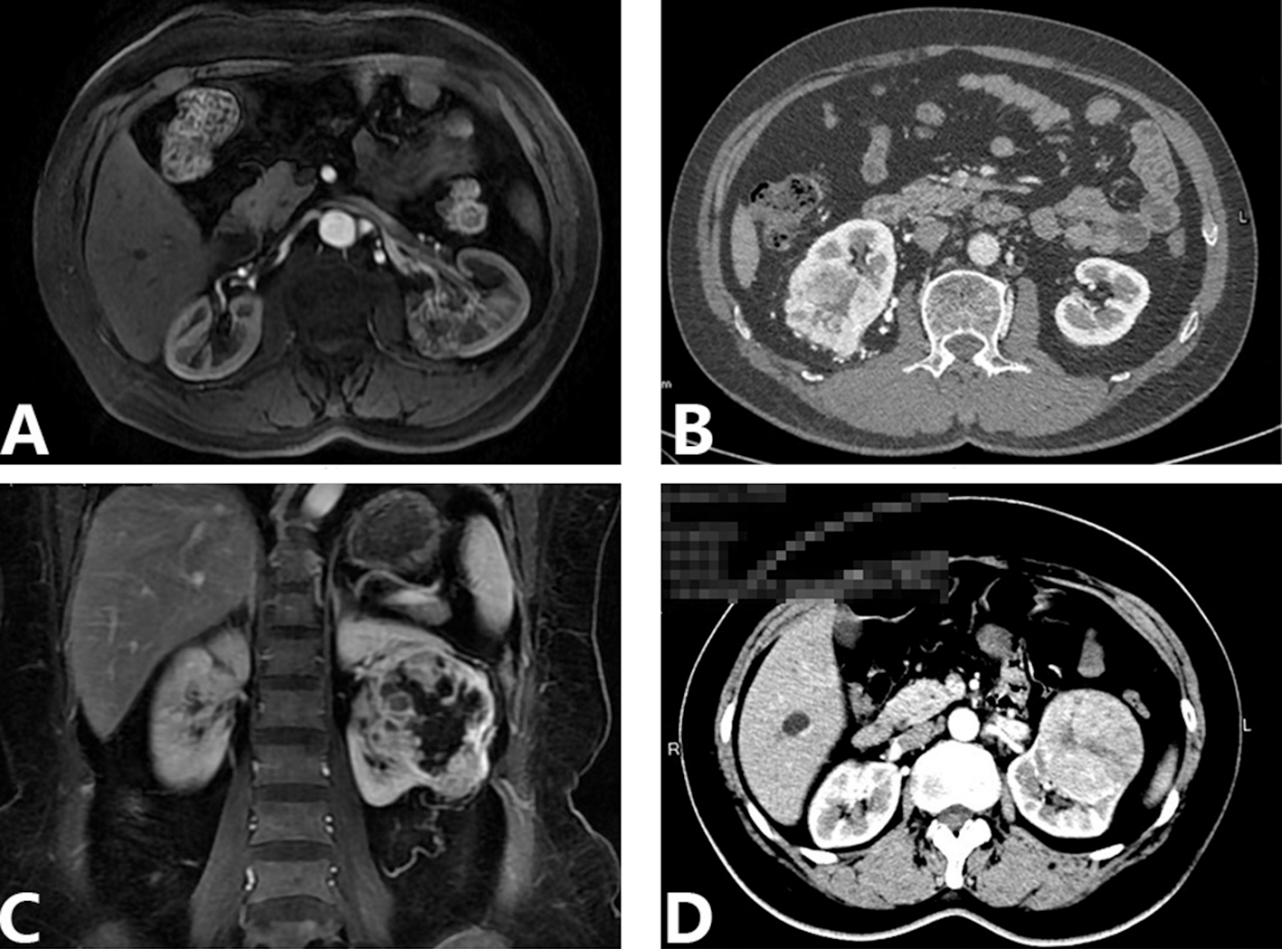
Axial or coronal corticomedullary phase contrast CT and MRI (**A**) A 63-year-old man with a renal mass in the left kidney. MRI revealed that the tumor extended into the sinus, with an irregular tumor shape and an ill-defined border between the tumor and sinus. This patient was stratified to the RSI high risk group and pathological reports demonstrated that ccRCC invaded into the renal sinus. (**B**) A 61-year-old man with a renal mass in the right kidney. CT revealed that the tumor was 6 cm in diameter, with necrosis in the mass and the tumor-perinephric fat border was ill-defined. This patient was stratified to PNI high risk group and pathological result identified ccRCC with PNI. (**C**) A 53-year-old woman with a renal mass in the left kidney. MRI showed that the tumor extended into renal sinus with an irregular tumor shape and necrosis in the center location. The diameter of the tumor was 7 cm and the margin was irregular on both the sinus and perinephric fat sides. This patient was stratified to the PNI and RSI high risk group and the pathological result revealed ccRCC with both PNI and RSI. (**D**) A 55-year-old woman with a renal tumor in the left kidney. CT showed that the renal tumor was 7 cm in diameter with a regular shape and tumor margin. This patient was stratified to the PNI and RSI low risk group and the pathological result demonstrated that ccRCC with no RSI or PNI. RSI: renal sinus invasion; PNI: perinephric fat invasion; CT: computed tomography; MRI: magnetic resonance imaging; ccRCC: clear cell renal cell carcinoma.

Further multivariate analysis identified tumor extension into the sinus (*p* = 0.031), an irregular tumor-sinus border (*p* < 0.001), and an irregular tumor shape (*p* = 0.028) as independent preoperative risk factors for RSI (Table 2, Figure 2A).

Patients were stratified according to risk factor patterns into low-, moderate-, or high-risk groups (Table 3). Subgroup analysis revealed that the RSI incidence was directly associated with the degree of preoperative risk (Kruskal-Wallis test, *p* < 0.001).

**Table 3:** Risk group classification based on preoperative risk factors for RSI

	Extend into sinus	Irregular border	Irregular shape	RSI %
Low risk	-	+-	+-	2.7% (4/149)
	+	-	-	
Moderate risk	+	+	-	16.1% (9/56)
	+	-	+	
High risk	+	+	+	51.6% (32/62)

RSI: renal sinus invasion.

Kruskal-wallis test, *p* < 0.001

### Risk factors associated with PNI

Univariate analysis revealed that large tumors (> 5 cm; *p* < 0.001), an irregular tumor-perinephric fat border (*p* < 0.001), tumor necrosis (*p* < 0.001), and an irregular tumor shape (*p* < 0.001) were preoperative factors associated with PNI (Table 4).

**Table 4:** Univariate and multivariate logistic regression analyses of risk factors for predicting PNI

Factors	Univariate analysis				Mutivariate analysis		
	HR	95% CI	*p*		HR	95% CI	*p*
SEX	1.034	0.434–2.460		0.939			
Age	0.997	0.963–1.031		0.839			
BMI	0.998	0.884–1.127		0.974			
Tumor size (cm)							
≤ 5	1				1		
> 5	8.510	3.120–23.213		< 0.001	4.824	1.459–15.945	0.010
Imaging extend to sinus							
No	1						
Yes	2.334	0.530–10.289		0.263			
Necrosis							
No	1				1		
Yes	7.571	2.744–20.893		< 0.001	3.281	1.099–9.794	0.033
Irregular border							
No	1				1		
Yes	0.092	0.030–0.276		< 0.001	0.203	0.057–0.729	0.015
Irregular shape							
No	1				1		
Yes	0.173	0.067–0.449		< 0.001	0.749	0.230–2.440	0.632

PNI, perinephric fat invasion; BMI, body mass index; HR, hazard ratio; CI, confidence interval.

Further multivariate analysis identified a tumor larger than 5 cm (*p* = 0.010), an irregular tumor-perinephric fat border (*p* = 0.015), and a tumor necrosis (*p* = 0.033) as independent preoperative risk factors for PNI (Table 4, Figure 2B).

Similarly, patients were stratified according to risk factor patterns into low-, moderate-, or high-risk groups (Table 5). Subgroup analysis revealed that PNI incidence was directly associated with the degree of preoperative risk (Kruskal-Wallis test, *p* < 0.001).

**Table 5:** Risk group classification based on preoperative risk factors for PNI

	Tumor > 5 cm	Irregular border	Tumor necrosis	PNI %
Low risk	**–**	**+**	**–**	1.2% (2/172)
	**+**	**–**	**–**	
	**–**	**–**	**+**	
	**–**	**–**	**–**	
Moderate risk	**+**	**–**	**+**	16.4% (9/55)
	**+**	**+**	**–**	
	**–**	**+**	**+**	
High risk	**+**	**+**	**+**	35% (14/40)

PNI, perinephric fat invasion.

Kruskal-wallis test, *p* < 0.001

## DISCUSSION

According to the 2009 Tumor-Node-Metastasis (TNM) staging system, a pT3a tumor included PNI, RSI, and invasion of the renal vein or its segmental branches [10]. However, RSI was identified as a principal route for advanced RCC development [9, 11]. Furthermore, Bonsib et al. [12] reported that > 90% of clear cell RCC tumors > 7 cm were associated with RSI. Thompson et al. [13] found that 67% of patients with pT1 tumors who died from the disease had undiagnosed RSI. In the present study, the RSI incidence was significantly greater than that of PNI and small renal vein invasion. Therefore, RSI was believed to be the primary cause of postoperative pathological upstaging, which would be congruent with previous studies [5-7]. Postoperative upstaging caused by renal vein invasion was not involved in the analysis because of the limited number of cases in this study.

Theoretically, RSI is more likely to result in metastasis and relapse because the renal sinus consists of lymphatics, and renal vein tributaries. Oncological outcomes associated with RSI have been reported previously in a meta-analysis that compared the cancer-specific survival (CSS) of patients with RSI and PNI across 6 studies comprising 1301 cases of T3a tumors. In that analysis, patients with RSI had significantly poorer CSS than those with PNI [14]. In addition, Timothy et al. [15] indicated that the combination of RSI plus PNI resulted in significantly poorer CSS than either RSI or PNI alone. At the present study endpoint, the recurrence rates were 21.2% (7/33) for RSI alone, 7.7% (1/13) for PNI alone, and 33.3% (4/12) for RSI plus PNI, although the differences between the groups were not significant. Similarly, Kaplan-Meier analysis showed that there were no significant differences in DFS between the groups.

Fernando et al. [16] reported that the positive surgical margin rate following pathological upstaging to pT3a was much higher than that in patients with pT1a tumors. Therefore, preoperatively assessing the possibility of RSI and PNI in patients with RCC could have a significant impact on treatment planning. The major blood vessels that supply the kidney pass through the renal sinus. Therefore, any tumors that extend into the renal sinus are often located close to major blood vessels. When NSS is used to treat such tumors, the tumor-sinus surgical margin is confined by these vessels, which must remain intact to supply blood to the kidney. For these reasons, RCC patients with RSI who have an incomplete pseudocapsule between the tumor and sinus and who undergo NSS are more likely to exhibit positive surgical margins. Regarding the patients with presumed PNI who undergo NSS, the perinephric fat tissue adjacent to the tumor needs to be dissected and removed together with the whole mass to avoid positive surgical margins. Alternatively, a RN procedure was a better surgical choice for these patients.

Several imaging studies to detect RSI in patients with RCC have been conducted. Bolster et al. [17] reported that tumor size, tumor location, an irregular tumor margin at the tumor-sinus border, and invasion to pelvicalyceal structures could aid a diagnosis of RSI. Kim et al. [18] showed that tumor size, an irregular tumor-sinus border, lymph node metastasis, and decreased perfusion of the diseased kidney were predictive risk factors for RSI. Sokhi et al. [19] concluded that tumor necrosis, an irregular tumor border, and direct contact between the tumor and sinus fat increased the risk of local invasion. The findings of the present study are congruent with those of these previous studies. Univariate analysis revealed 5 potential risk factors for RSI, 3 of which remained significant in the multivariate analyses. Among these 3 risk factors, tumor extension into the sinus was essential for RSI, whereas an irregular tumor-sinus border and irregular tumor shape reflected the malignant characteristics of the tumor. Therefore these 3 risk factors might be reliable predictors of RSI, even for micro-RSI, which is difficult to detect using current imaging technology.

Detection of PNI on computed tomography (CT) and magnetic resonance imaging (MRI) is sometimes difficult because of other pathologic process such as inflammation, hematoma, and abscesses and so on. Studies concerning PNI were also reported recently. Kim et al concluded that multidetector computed tomography (MDCT) shows a relatively high diagnostic performance in detecting PNI of RCC. Tumor size, fat infiltration with a nodular appearance, and an irregular tumor margin were predictors for PNI [20]. Whereas Landman et al found that perinephric soft-tissue standing was shown to be the only significant factor for predicting PNI in tumors 4 cm or less [21]. Using the same analysis methods as for RSI, we identified independent risk factors for PNI as follows: tumor larger than 5 cm, an irregular tumor- perinephric fat border, and a tumor necrosis. These CT or MRI signs were illustrated in Figure 2.

In the present study, patients were stratified into risk groups according to the preoperative presence or absence of the independent risk factors. We would suggest that, for patients categorized as low-risk (Figure 2D), the NSS procedure could be performed without compromising oncological safety. In contrast, for patients categorized as moderate- or high-risk (Figure 2A–2C), the RN procedure would be more suitable. However, if an indication for NSS exists, such as a solitary kidney, then the surgical margin at the tumor border should be evaluated with extreme caution, with long-term postoperative follow-up and surveillance.

The present study had limitations. First, the study was retrospective. Second, the present study only evaluated patients who underwent RN because the kidney, perinephric fat, and renal sinus are removed in RN specimens, thereby enabling precise pathological upstaging assessment. However, RN is reserved for tumors considered as advanced or untreatable local RCC. Both of these limitations indicate definite selection bias and consequent stage migration. Third, the sample size was small, and the follow-up duration was short, which could have affected the data regarding DFS. Finally, the findings in the present study refer to RSI and PNI, and cases including renal vein or pelvicalyceal system invasion were not included. Therefore, large, prospective, long-term follow-up studies are needed in patients with different types of invasion to validate our findings.

In conclusion, postoperative upstaging is not rare in RCC. Preoperative imaging data obtained using MRI or MDCT might be useful for identifying the upstaging risk, which could have implications for patient outcomes. More specifically, tumor extension into the sinus, an irregular tumor-sinus border, and an irregular tumor shape were preoperative risk factors associated with the RSI. And a tumor larger than 5 cm, an irregular tumor-perinephric fat border, and a tumor necrosis were preoperative risk factors associated with the PNI. Patients could receive improved preoperative counseling and treatment planning based on an assessment incorporating these risk factors.

## MATERIALS AND METHODS

This retrospective study was approved by the ethical committee of our institution. Clinicopathological and imaging data from 300 consecutive patients with RCC who underwent RN between March 2016 and January 2017 were reviewed. Imaging data for all patients was obtained using MRI and MDCT and was independently evaluated and summarized by 2 reviewers who were blinded to the clinicopathological data. In the event of any discrepancies, the reviewers re-evaluated the images together until a consensus was reached. The definitions of the risk factors from the CT/MRI imaging were as follows: tumor size was defined as the largest diameter of the tumor assessed in the imaging information. Imaging extend to sinus was defined as the tumor direct contact with or bulge into the renal sinus in the imaging. Necrosis on MRI was defined as high signal intensity on T2-weighted images (but still lower than fluid), low signal intensity on T1-weighted images, lack of enhancement, and central location within the tumor [22]; and necrosis on CT was defined as a non-enhancing, low-attenuating lesions of less than 20 Hounsfield Unit (HU) on unenhanced scans with ill-defined or irregular margins [23]. Irregular border in RSI assessment was defined as the presence of an irregular or ill-defined tumor margin at the tumor-renal sinus interface; and irregular border in PNI assessment was defined as the presence of an irregular or ill-defined tumor margin at the tumor-perinephric fat interface, perinephric soft-tissue stranding, or perinephric contrast-enhancing soft-tissue nodules. Irregular shape was defined as the presence of multiple lobes or nodules. Based on the pathological and imaging data, 7 patients with pT3b tumors, 1 with a pT3c tumor, 1 with a pT4 tumor, 5 with multilocular cystic renal cancer, and 1 with a Wilms’ tumor were excluded. Furthermore, 18 patients for whom imaging data were unavailable were excluded. None of the patients had preoperative metastases. The final analysis cohort included 267 patients with RCC.

RSI and PNI were defined as described previously [7]. Pathological data were confirmed by a pathologist with 20 years of accumulated experience. Patients were followed-up for a median of 7.5 months (range, 3.6–14.8 months), with monitoring for postoperative metastases or recurrence. Tumor stage was classified according to the 2009 TNM staging system [10]. Tumor grade was determined histopathologically according to Fuhrman’s classification [24]. After the independent risk factors were confirmed, patients were stratified into low-, moderate-, or high-risk groups according to the preoperative presence or absence of these factors. For RSI risk group classification, patients without tumor extend into sinus were stratified to the low risk group, no matter the other two risk factors presented or not. Patients with tumor extend into sinus and one of the other two risk factors were stratified to the moderate risk group. And patients with all the risk factors were stratified to the high risk group. For PNI risk group classification, patients with none or only one risk factors were stratified to the low risk group. Patients with two risk factors were stratified to the moderate risk group. And patients with all the risk factors were stratified to the high risk group.

### Statistical analyses

Data were presented as the mean ± SD for continuous variables and number (percentage) for categorical variables. Univariate and multivariate logistic analyses were used to identify risk factors associated with RSI and PNI. Variables with a *p*-value of < 0.05 in the univariate analysis were included in multivariate analysis. DFS was compared using Kaplan-Meier analysis with the log-rank test. Risk group classification was evaluated using the Kruskal-Wallis test. A two-tailed *p*-value of < 0.05 was considered statistically significant.

## Abbreviations

RCC: renal cell carcinoma
RSI: renal sinus invasion
PNI: perinephric fat invasion
NSS: nephron-sparing surgery
RN: radical nephrectomy
DFS: disease free survival
CSS: cancer specific survival
CT: computed tomography
MDCT: multidetector computed tomography
MRI: magnetic resonance imaging
